# Cystotomy for Cystic Calculus

**Published:** 1899-06

**Authors:** 


### CLINICAL REPORTS.

### VETERINARY HOSPITAL, UNIVERSITY OF PENNSYLVANIA.

CLINIC OF S. J. J. HARGER, V.M.D.,
PROFESSOR OF VETERINARY ANATOMY AND ZOÖTECHNICS.

CYSTOTOMY FOR CYSTIC CALCULUS. The patient was a cocker spaniel bitch, received April 7, 1899.

*History.* Three months ago the bitch was seen to discharge some blood with the urine. This was not observed again until about a week before entering the hospital.

*Symptoms.* General condition good. Frequent micturition, the urine escaping in small quantities and tinged with blood; also slight incontinence of urine. Wherever the bitch sat down a wet spot was left. At times there also was considerable straining, with no urinary discharge. Palpation over the abdomen revealed a hard, round, movable tumor opposite to the flank, whose forward displacement was limited, but which could be pushed into the pelvic cavity. Calculus of the bladder was suspected after exclusion of other conditions, and the diagnosis was confirmed by passing a metallic sound into the bladder and locating the stone.

*Treatment.* Efforts at crushing the calculus with a lithotrite were made, but could not be effected. Cystotomy—incising the bladder after laparotomy—was the only remaining available resource. A purgative dose of castor-oil was administered two days before, in order to empty the bowels. The abdominal surface was prepared in the usual manner and median laparotomy performed; the incision, about two and one-half inches in length, terminated behind, close to the brim of the pelvis. The patient was etherized.

The bladder, which was considerably elongated, was drawn out through the abdominal incision and a clean towel wrapped around its neck to prevent the entrance of blood into the peritoneal cavity. The fundus of the bladder was turned backward, so as to expose its superior surface. A median incision about two inches long was made over this surface. The stone thus exposed was extracted. After arresting the hemorrhage and removing all clots from the interior of the bladder, the incision was closed with a continuous catgut suture. The external surface was cleansed with a bichloride solution, and the organ was replaced into the abdominal cavity. The external incision was treated in the usual manner.

The bladder was the seat of marked cystitis, all its tunics being thickened, and the hemorrhage was considerable. The calculus, spherical in form, measured one and one-half inches in diameter and weighed one and three-quarter ounces.

A favorable prognosis was scarcely anticipated, although happily the animal made a good recovery. The appetite remained fair and the temperature never rose above 103.7° F. At first there was some evidence of abdominal pain, but the animal gradually improved, and the act of micturition became normal. For ten days after the operation an emulsion of balsam of copaiba and fluid extract of uva ursi was administered. At the end of three weeks the patient was discharged.

